# Utilization of focused antenatal care among expectant women in Murang'a County, Kenya

**DOI:** 10.11604/pamj.2021.39.23.26339

**Published:** 2021-05-10

**Authors:** Kiplangat Titus Mutai, George Ochieng Otieno

**Affiliations:** 1 School of Public Health and Applied Human Sciences, Kenyatta University, Nairobi, Kenya,; 2 Department of Health Management and Informatics, Kenyatta University, Nairobi, Kenya

**Keywords:** Focused antenatal care, utilization, Gatanga, Kenya

## Abstract

Focused Antenatal Care (FANC) is crucial to improving maternal and infant health. Despite the Government of Kenya' efforts to reduce maternal and neonatal morbidities and mortalities, these conditions prevail in Murunga. The current study examined how individual, organizational, and policy factors influence the utilization of focused antenatal care services amongst women in the Gatanga sub-county, Murang'a County, Kenya. The cross-sectional survey data was collected between June and July 2019 from three sampled wards. A structured questionnaire was administered to 334 women of reproductive age, aged 18 years and above, who delivered within the past one year or above 38 weeks of gestation. Descriptive statistics and chi-square tests at a 5% level of significance were done using SPSS version 22. The findings indicated that 37.3% of respondents do not utilize FANC services. Level of education (X^2^ (3) = 16.05; p < 0.05), occupation (X^2^ (3) = 16.50; p < 0.05), level of income (X^2^ (4) = 15.53; p < 0.05), time taken to the facility (X^2^ (3) = 34.72; p < 0.05), and waiting time (X^2^ (3) = 14.17; p < 0.05) were found to significantly influence utilization of FANC services. Therefore, women should be empowered through education and economic activities to remain financially independent. The government should also improve access to health care, especially in rural areas, by building new health facilities to improve the utilization of FANC services. Besides, more health care providers should be employed to reduce the waiting time at the facility.

## Introduction

Pregnancy-associated complications contribute to more than half the deaths among women annually, with 90-95% from developing countries [1]. Globally, approximately 86% of expectant women attend antenatal healthcare facility at least once, while 62% meet the recommended four visits [2]. Antenatal care (ANC) is crucial in preventing maternal and fetal mortality and morbidity [3]. More than 95% of women make ANC visits in developed countries hence recording low maternal and neonatal morbidities and mortalities. The World Health Organization [4] promoted the adoption of a new ANC model targeting low-income countries. The model is referred to as 'focused' ANC, consisting of at least four visits to a health facility during pregnancy [4]. The revised model tended towards reduced but goal-orientated clinic visits [5]. Focused antenatal care (FANC) is an individualized service provided to an expectant mother that emphasizes overall health, preparation for childbirth, and prevention of possible pregnancy and birth-related complications [1]. Focused antenatal care, alongside family planning practices, skillful delivery, and emergency obstetrical care, is a fundamental package of services that improve maternal and newborn health [5].

Focused antenatal care is essential in maternal safety. It improves maternal and neonatal outcomes [6]. The goal of FANC is to reduce delays at service provision sites, improve quality of service, and advocate for skilled birth attendance [7,8]. Focused antenatal care reduces the cost of care in developing countries by reducing the number of visits for low-risk pregnancies. FANC emphasizes the quality of visits, personalized care, early disease diagnosis, and birth-related complications readiness rather than the number of clinic visits [9]. During the visits, expectant women are educated on breastfeeding, family planning options, and nutritional care. They also receive tetanus toxoid injection [10]. The necessary antenatal profile done includes tests for haemoglobin level, HIV, syphilis, urinary tract infections, and blood grouping [1]. It is recommended that a woman attend antenatal care at least four times during pregnancy [1]. The recommended FANC visits are as follows: before 16 weeks, between 16 to 28 weeks, between 28-32 weeks, and at 36 weeks. However, surveys from sub-Saharan Africa have shown that women often utilize ANC services after the first trimester and hence do not reach the recommended number of FANC visits [6]. The utilization of FANC aims to ensure the good health of the mother and the newborn baby by early detection and management of complications and preparedness for any problem during labour progress. Less utilization of ANC could increase prenatal fetor-maternal complications [11]. Additionally, reduced ANC utilization predisposes babies to low birth weight [12].

Kenya has adopted the World Health Organization (WHO) goal-oriented antenatal care package and designed new guidelines focusing on ANC, birth preparedness for emergencies, diagnosis, and treatment of life-threatening conditions during the antenatal and perinatal period [13]. The intervention ensures that every pregnant woman and newborn is accorded good care during pregnancy. Despite the efforts to curb mortality, less than half of expectant women in Kenya meet the recommended four ANC visits [14]. Murang'a County registered a high maternal mortality rate despite various spirited interventions to reduce maternal mortality recorded to be 350 per 100000 live births with Millennium Development Goals (MDGs) status at National being 488 [15]. Deliveries by skilled health attendant were 54%, with a maternal mortality of 107/100,000 live births in 2018 [16]. The FANC attendance at the first visit in Murang'a County is much lower than the national level (62%), with 27% of the women completing the recommended four visits. The proportion is lower than in Tharaka Nithi County, Kenya, with 52% utilization of FANC [10]. In this regard, this study assessed how individual, organizational, and policy factors influence the utilization of focused antenatal care services in the Gatanga sub-county.

## Methods

**Study design:** the study employed a cross-sectional study design conducted in the Gatanga sub-county, Murang´a County. The 334 study participants who meet the inclusion criteria were spread across the three randomly selected wards of Ithanga (101, 30.6%), Mitubiri (120, 36.4%), and Gatanga 109 (33.0%). The data was collected using a questionnaire between June and July 2019. Descriptive analysis was conducted followed by Pearson´s Chi-square test of association between selected predictors and measures of utilization of FANC.

**Setting:** the target population consisted of all women of reproductive age (15-49 years) in the Gatanga sub-county, Murang´a County. The location was chosen since the utilization of the recommended four FANC visits in Murang´a county (27%) [16] is much lower than the national level (58%) [17]. The study sorted to determine the extent of Murang´a county integrated development plan (2018-2022) to increase skilled birth attendance and utilization of focused antenatal care. Gatanga sub-county comprises five-county administrative wards: Ithanga ward, Kakuzi/Mitubiri ward, Kihumbuini ward, Gatanga ward, and Kariara ward.

**Participants:** multistage sampling was applied to recruit the 334 study participants aged between 18 to 49 years and must have delivered within the past one year or above 38 weeks of gestation. Women with abnormal pregnancies were not eligible for inclusion in the study. Additionally, women who migrated to this region within one year or otherwise received service outside the study area were not considered. First, simple random sampling was used to select three wards and three community units in each ward. Secondly, systematic sampling (k=6) was used to select households at the community units. The first household was randomly picked, followed by every 6^th^ household until the quota was reached. A household was selected if a household member met the inclusion criteria otherwise, skipped. If more than one woman of reproductive age lived together in a household, a simple random sampling technique was used to pick one respondent.

### Variables

**Outcome variables:** this study dichotomised the utilisation of FANC into two sub-variables. First, we considered utilising the FANC package with two responses as either “utilised” if the women met the required four or more recommended FANC visits and “not utilised” for those who attended less than four FANC visits for their most recent pregnancy. Secondly, we considered the frequency of visits with four levels: 0 - 3 and 4 or more times.

### Predictor variables

#### Individual factors

**Age:** maternal age at birth in completed years. The variable was categorical with five levels: below 22 years, 23-27 years, 28-32 years, 33-37 years, and above 37 years.

**Education level:** no formal education, primary education, secondary or post-secondary education.

**Marital status:** either married, separated, divorced, engaged or single.

**Occupation:** four levels including formal employee, self-employed, casual labor or housewife.

**Income level:** refers to the monthly income level and was categorized into; less than Ksh 5000, 5000-10 000, 10 001-20 000, 20 001-50 000 more than Ksh 50 000.

**Parity:** refers to the number of live children born to a woman. The variable was categorized into four levels: 1, 2, 3, or 4 and more births.

### Institutional factors

**Time taken to reach the health facility:** measured in total hours taken by the time taken by women from their homes to the health care facility offer antenatal care services. The variable had four levels: within an hour, half of an hour to 1 hour, 1-2 hours, and above 2 hours.

**Missed service:** measures whether a woman received or missed a complete antenatal care package at the end of their FANC visits or not.

**Waiting time:** time taken in the queue before receiving the antenatal services.

**The attitude of clients to health facilities:** clients´ willingness to recommend to someone else to visit ANC unit after her experience. The variable assumes two levels: yes or no.

### Policy factors

**Cost of FANC services:** include fees or charges women receive when they visit the health care facility for antenatal care services.

**Source of funds:** source of funds used to cater for antenatal care services which can either be from savings, borrowing, Linda Mama card, insurance, or free services.

**Ownership of Linda Mama card:** asks whether a woman owns the Linda Mama Card hence has two levels: yes or no.

**Benefits of Linda Mama program:** asks whether the Linda Mama program covers women when they receive the FANC services hence has two levels: yes or no.

**Data collection technique:** a questionnaire was used to collect the required information from the respondents over the period between June and July 2019.

**Bias:** questionnaire bias was minimized before and after the study. First, the instruments were well-reviewed to ensure that the level of each categorical variables was free from faulty scale with no overlapping intervals. During the administration of the questionnaires, the selected enumerators who had medical background were trained and familiarized with the various terminologies and definitions used in the study and how to collect the required data from households. The interviewer training also helped eliminate semantic bias, given the educational level diversity of the respondents.

**Sample size determination:** probability proportionate to size sampling methodology was used as specified by Fischer 1998. The sample size n was obtained using the following formula;

$$n=\left(\frac{Z^{2}pq}{d^{2}}\right)$$

(1) Where: n is the sample size if the target population is more than 10,000; z is the standard normal deviate at the required confidence level; p is the proportion of women currently utilizing complete FANC package; q = (1- p); d is the desired level of precision. Given that the proportion of women attending four focused antenatal care visits in Murang'a County is 27% [16], n = (1.96^2^ × 0.27 × 0.73)/0.05^2^) = 303. The study added 10% of the sample size to care for non-responses. Thus, the sample size used in the study was 303 + 31 = 334 respondents.

**Quantitative variables:** waiting time, in hours, was measured on a continuous scale. Yet, the test of independence using the chi-square test requires two categorical variables. Thus, the variable was categorized into four levels: within an hour, half of an hour to 1 hour, 1 to 2 hours, and above 2 hours. Similarly, the cost of FANC services was categorized into 350 and below, between 351 and 700, between 701 and 1500, and above 1500.

**Data analysis techniques:** the data was analyzed using Statistical Package for Social Sciences (SPSS) version 22. Descriptive statistics and Chi-square test at a 5% level of significance were used to test the association of the stated factors and the utilization of FANC.

**Ethical considerations:** the proposal was approved by the Kenyatta University Graduate School Ethical and Research Committee. A research permit was then obtained from the National Commission for Science, Technology, and Innovation (NACOSTI) before the commencement of the study. Permission and approval to carry out the household survey were obtained from the Murang´a county health department and the community leadership. Informed consent was also obtained orally from the study participants. Assured confidentiality of information gathered the study's participation was purely a voluntary process with no coercive methods or payoff to influence the participants.

## Results

Out of the administered questionnaires, 330 were completed making the response rate 98.8%. The distribution of the sample size by ward was 101 (30.6%) in Ithanga, 120 (36.4%) in Mitubiri, and 109 (33%) in Gatanga.

**Socio-demographic characteristics of the study population:** the majority of the respondents in the Gatanga sub-county were aged below 22 years (30.6%). Those aged above 37 years had the least proportion (7.6%). Majority of the respondents acquired primary education with a percentage of 49.1%. Only 0.9% did not complete their primary education and represented the least proportion of respondents. Other summary statistics are shown in Table 1.

**Table 1:** association between demographic characteristics with the utilization of FANC

	Distribution		Utilization of FANC				χ^2^_a_	df	p
			Yes		No				
	N (330)	%	n (207)	%	n (123)	%			
**Age in years**									
< 22	101	30.6	66	65.3	35	34.7			
23 - 27	87	26.4	53	60.9	34	39.1			
28 - 32	70	21.2	39	55.7	31	44.3			
33 - 37	47	14.2	31	66	16	34			
> 37	25	7.6	18	72	7	28			
**Level of education**									
No schooling	3	0.90	1	33.3	2	66.7	16.05	3	0.001***
Primary	162	49.1	110	67.9	52	32.1			
Secondary	143	43.3	76	53.1	67	46.9			
Post-secondary	22	6.7	20	90.9	2	9.1			
**Occupation**									
Formal employee	21	6.4	18	85.7	3	14.3	16.50	3	0.001***
Self employed	69	20.9	54	78.3	15	21.7			
Casual labour	80	24.2	43	53.8	37	46.2			
House wife	160	48.5	92	57.5	68	42.5			
**Monthly income**									
< 5000	218	66.1	125	57.3	93	42.7	15.53	4	0.004***
5000-10000	51	15.5	31	60.8	20	39.2			
10000-20000	39	11.8	33	84.6	6	15.4			
20000-50000	17	5.2	15	88.2	2	11.8			
> 50000	5	1.5	3	60	2	40			
**Marital status**									
Married	246	74.5	160	65	86	35	3.94	4	0.414
Separated	13	3.9	6	46.2	7	53.8			
Divorced	3	0.9	1	33.3	2	66.7			
Engaged	8	2.4	4	50	4	50			
Single	60	18.2	36	60	24	40			
**Parity**									
1	87	26.4	53	60.9	34	39.1	1.26	3	0.739
2	92	27.9	57	62	35	38.0			
3	87	26.4	53	60.90	34	39.1			
4 or more	64	19.4	44	68.80	20	31.2			

Note: all percentages are row percentages; a Chi-square; ***significant at 1% level; df: degrees of freedom

**Frequency of attendance:** the findings indicated 73 (35.3%) of the 207 respondents attended the health facility fully completed the recommended number of visits (Table 1). One hundred and twenty-three participants (37.3%) did not make any clinical visit for FANC services.

**Individual factors affecting utilization of FANC services:** the findings indicated that level of education (X^2^ (3) = 16.05; p < 0.05), occupation (X^2^ (3) =16.50; p < 0.05), and level of income (X^2^ (4) = 15.53; p < 0.05) significantly influences utilization of FANC. However, age (X^2^ (4) =3.02; p>0.05), marital status (X^2^ (4) = 3.94; p >0.05), and parity (X^2^ (3) = 1.29; p>0.05) did not significantly influence the utilization of FANC services (Table 1). The significant demographic factors were further correlated with frequency of attendance. Chi-Square test results indicate that level of education (X^2^ (12) = 35.06; p < 0.05), occupation (X^2^ (12) = 24.56; p < 0.05), and monthly income (X^2^ (16) = 36.66; p < 0.05) significantly influences the frequency of attendance (Table 2).

**Table 2:** demographic characteristics associated with the frequency of utilization of FANC

	Number of times attended										χ^2^_a_	df	P
	0		1		2		3		4 or more				
	n (123)	%	n (14)	%	n (31)	%	n (89)	%	n (73)	%			
**Level of education**													
No schooling	2	66.7	0	0	0	0	1	33.3	0	0	35.06	12	0.000**
Primary	52	32.1	5	3.1	19	11.7	57	35.2	29	17.9			
Secondary	67	46.9	8	5.6	9	6.3	27	18.9	32	22.4			
Post – secondary	2	9.1	1	4.5	3	13.6	4	18.2	12	54.5			
**Occupation**													
Formal employee	3	14.3	0	0	3	14.3	8	38.1	7	33.3	24.56	12	0.017*
Self employed	15	21.7	5	7.2	11	15.9	23	33.3	15	21.7			
Casual labour	37	46.2	1	1.2	5	6.2	22	27.5	15	18.8			
House wife	68	42.5	8	5	12	7.5	36	22.5	36	22.5			
**Monthly income**													
< 5000	93	42.7	6	2.8	18	8.3	64	29.4	37	17	36.66	16	0.002**
5000 -10000	20	39.2	3	5.9	7	13.7	7	13.7	14	27.5			
10000 -20000	6	15.4	2	5.1	5	12.8	10	25.6	16	41			
20000 -50000	2	11.8	3	17.6	1	5.9	7	41.2	4	23.5			
> 50000	2	40	0	0	0	0	1	20	2	40			

a Chi-square; df: degrees of freedom; p: p-value, ***significant at 1% level; *significant at 5% level

**Organizational factors influencing utilization of FANC services:** the average time taken to reach the health facility was found to be approximately 1 hour 2 minutes with a standard deviation of 0.758. The mean waiting time was found to be 0.84 hours which is approximately 50 minutes. The chi-square test of association indicated that utilization of FANC services is significantly dependent on the time taken to reach the health facility (X^2^ (3) = 34.72; p< 0.05) and waiting time and waiting time (X^2^ (3) = 14.17; p < 0.05) (Table 3). The two significant variables were further correlated with the frequency of attendance. Frequency of attendance was also found to significantly depend on time taken to reach the facility (X^2^ (3) = 42.08; p < 0.05) and waiting time (X^2^ (12) = 29.32; p < 0.05) (Table 4).

**Table 3:** measures of association between organizational factors and the utilization of FANC

	Utilization of FANC				χ^2^_a_	df	p
	Yes		No				
	n (207)	%	n (123)	%			
**Time taken to reach the facility in hours**							
Less than half of an hour	87	77.7	25	22.3	34.73	3	0.000***
Half of an hour to 1 hour	85	66.9	42	33.1			
1 to 2 hours	25	39.7	38	60.3			
Above 2 hours	10	35.7	18	64.3			
**Waiting time at the facility in hours**							
Within half of an hour	119	68.8	54	31.2	14.17	3	0.003***
1/2 to 1 hour	64	62.7	38	37.3			
Between 1 and 2 hours	19	51.4	18	48.6			
More than 2 hours	5	27.8	13	72.2			

a Chi-square; df: degrees of freedom; p: p-value, ***significant at 1% level

**Table 4:** organizational factors associated with frequency of utilization of FANC

	Number of times attended										χ^2^_a_	df	P
	0		1		2		3		4 or more				
	n (123)	%	n (14)	%	n (31)	%	n (89)	%	n (73)	%			
**Time taken to the health facility**													
<½	25	22.3	5	4.5	16	14.3	33	29.5	33	29.5	42.08	3	0.000***
½ - 1	42	33.1	6	4.7	8	6.3	39	30.7	32	25.2			
2-Jan	38	60.3	3	4.8	4	6.3	13	20.6	5	7.9			
>2	18	64.3	0	0	3	10.7	4	14.3	3	10.7			
**Waiting time at the facility**													
<1/2	54	31.2	5	6.4	11	5.8	53	30.6	45	26	29.32	12	0.004***
½ - 1	38	37.3	2	2	16	15.7	27	26.5	19	18.6			
2-Jan	18	48.6	1	2.7	4	10.8	5	13.5	9	24.3			
> 2	13	72.2	0	0	1	5.6	4	22.2	0	0			

a Chi-square; df: degrees of freedom; p: p-value; ***significant at 1% level

**Policy factors influencing utilization of FANC:** the cost and utilization of FANC services was found to statistically independent (X^2^ (3) = 0.425; p > 0.05) (Table 5). The findings indicated that 85.5% of women who attended the clinic received FANC services. The attitude was measured by the clients' willingness to recommend to someone else to visit ANC unit after her experience. Ninety-point nine percent of the women recommend one to visit the ANC facility. Most women (68%) benefit from the free FANC services provide at the visited health facility. Additionally, 20.6% of the women benefited from the Linda Mama Programme. Linda Mama programme penetration was found to be high. Seventy-four-point two percent (74.2%) ownership of cards is a good proportion with only 23.3% of the respondents without the Linda mama card (Table 6).

**Table 5:** association of cost of antenatal care services with the utilization of FANC

Amount paid for service (Ksh.)	Utilization of FANC				χ^2^_a_	df	p
	Yes		No				
	n (207)	%	n (123)	%			
≤ 350	192	62.7	142	37.3	0.425	3	0.935
351-700	5	55.6	4	44.4			
701-1500	5	71.4	2	28.6			
>1500	5	62.5	3	37.5			

a Chi-square; df: degrees of freedom; p: p-value

**Table 6:** policy factors influencing the utilization of FANC

	Frequency(n)	Percentage (%)
	n (207)	
**Missed services^1^**		
Yes	30	14.5
No	177	85.5
	n (330)	
**Will you recommend someone else to the facility?**		
Yes	300	90.9
No	30	9.1
**Source of funds**		
Savings	28	8.5
Borrowed	8	2.4
Linda Mama	68	20.6
Insurance	8	2.4
Free service	218	66.1
**Ownership of Linda Mama card**		
Yes	245	74.2
No	77	23.3
Never heard	8	2.4

1 is directed to those who attended the clinic

## Discussion

The proportion of attendance was above average (62.7%) which is higher than the national level (58%). Those who completed the recommended number of visits were 21.1%. This proportion is smaller than the report by [16], which indicated that 27% of women make four FANC visits. However, with the government's effort to provide free and affordable maternity care services in addition to the Linda mama program aimed at providing a package of essential health services as a waiver of user fee at National Level and improving access to healthcare to all Kenyans, the proportion of nonattendance should be close to zero.

**Individual factors:** the study results indicated that women's education level, occupation, and income level significantly influenced FANC utilization and the frequency of attendance to the ANC units. The study findings are similar to those of [3,18,19]. Lack of formal education and lower education level is associated with low FANC visits amongst women. Education positively impacts health-seeking behaviours by exposing women to more health-related education information, making them fully aware of the risks and complications associated with avoiding FANC services [20]. Additionally, education can be seen as playing a significant role in promoting women´s eligibility for employment, raising their income levels. With high-income levels, the indirect cost associated with utilization of FANC, such as the cost of transport, is not problematic to them. Concerning occupation, women who are employed are more likely to attend at least four ANC visits than the unemployed. Thus, to increase the utilization of FANC, unemployment issues have to be addressed. According to [6], attending a FANC facility involves incurring indirect costs such as travel costs and food. With most respondents earning less than Ks 5,000 per month (66.1%), there is a high level of dependency among the women in reproductive age in Gatanga sub-county. Thus, this can be a limitation to free access to maternal care requirements owing to unavoidable indirect costs. The low-income levels can be attributed to low levels of education since such women are not eligible for formal employments with higher income levels. Moreover, the findings indicated that higher-income earners had better utilization of FANC services than low-income earners. This can be attributed to the cost of transport to the health facility. Similar results were found in Mwingi, Kenya by [21]. Additionally, the underutilization of ANC services in Indonesia was linked to a wealth index [22].

**Organizational factors:** time taken to reach the facility and utilization of FANC were found to be statistically dependent. Women residing far away from the healthcare facility were more likely to utilize the FANC services. This is because walking or travelling for long hours could be tiresome to pregnant women. Moreover, distance-related costs may discourage them from visiting ANC services. It can also be attributed to the perceived loss of working time. Similar findings have been found by [23,24]. Waiting time was also found significantly influence utilization of FANC. Similar results were found in Indonesia by [25]. In essence, long waiting times are generally tiresome hence may discourage women from making subsequent visits to the FANC unit. The likelihood of a woman attending the FANC unit to recommend someone else to visit the clinic depends on whether the services provided are satisfactory. According to [26], poor communication between patients and healthcare providers, unfriendly behavior, and negative attitudes towards the healthcare providers hinder women from receiving healthcare services. The findings indicated that 90.9% of women can recommend one to visit the FANC facility. This is indicative that women in Gatanga sub-county have a positive attitude towards the FANC services and the service providers.

**Policy factors:** the research established that the majority (92.7%) of the women in Gatanga sub-county who were charged to pay for FANC pay not more than Ksh 350. Only 2.4% pay above Ksh 1,500. Thus, the services are affordable to most of the women in need of FANC services. It is an indication that Kenya's government initiative to promote free and/or affordable maternity services has been, to a greater extend, adopted in Gatanga sub-county. The free services should act as an incentive towards the promotion of the utilization of FANC services in a bid to address antenatal and post-natal mortality rates. The cost of FANC was also found have a significant influence on the utilization of FANC services. A majority of the women (66.1%) benefited from the free ANC services, with 20.6% benefiting from the Linda Mama program. The health insurance package provides a package of basic health services aimed at waiving user fees at the national level and improving access to healthcare to all Kenyans expanding its coverage, both private and faith-based facilities. The two programs, being publicly funded health schemes, have ensured that pregnant women and infants have access to quality and affordable health services.

## Conclusion

The findings revealed that Kenya's government initiative to promote free and/or affordable maternity services has been, to a greater extend, been adopted in Gatanga sub-county. Most of the women receive free FANC services. However, the proportion of women meeting recommended four FANC visits and above remains low (21.1%). Besides, the extent to which women with ownership of Linda Mama card has penetrated in this sub-county is good (74.2% ownership). Therefore, women should be encouraged to attend the clinic to receive these free services to address challenges such as complications during birth and neonatal mortality. Women should be empowered through education and participation in economic activities to give them financial independence. Besides, the legal framework should reduce early marriages. Through the ministry of education, the government should ensure high female school enrollment and transition rates. The government of Kenya should also improve access to health care, especially in rural areas, by building new health facilities. Moreover, more medical practitioners should be employed to reduce the waiting time at the health facility. If these aspects are addressed, the hundred per cent utilization of the four visits of the FANC services, as recommended by WHO, will be achieved.
